# Lanostane Triterpenoids from the Fruiting Bodies of *Fomes officinalis* and Their Anti-Inflammatory Activities

**DOI:** 10.3390/molecules25204807

**Published:** 2020-10-19

**Authors:** Jianxin Han, Wei Liu, Miaomiao Li, Yanpei Gu, Ying Zhang, Tao Yuan

**Affiliations:** 1 Department of Food Science and Nutrition, School of Biosystems Engineering and Food Science, Zhejiang Key Laboratory for Agro-Food Processing, Zhejiang University, Hangzhou 310058, China; jianxin@zju.edu.cn (J.H.); happygyp@126.com (Y.G.); 2 The Key Laboratory of Plant Resources and Chemistry of Arid Zone, and State Key Laboratory of Xinjiang Indigenous Medicinal Plants Resource Utilization, Xinjiang Technical Institute of Physics and Chemistry, Chinese Academy of Sciences, Urumqi 830011, China; ucasliuwei@126.com; 3 The Laboratory of Effective Substances of Jiangxi Genuine Medicinal Materials, College of Life Sciences, Jiangxi Normal University, Nanchang 330022, China; jxnulmm@163.com

**Keywords:** *Fomes officinalis*, polyporaceae, lanostane triterpenoid, spirostructure, anti-inflammatory

## Abstract

In the current study, further chemical investigation of the fruiting bodies of *Fomes officinalis* led to isolate seven new 24-methyl-lanostane triterpenoids, named officimalonic acids I−O (**1**–**7**). Their structures were elucidated based on the analysis of spectroscopic data (HR-MS, 1D and 2D NMR, UV, IR). Compounds **1**−**3** possessed an unusual C-23 spirostructure moiety, while compounds **4**−**7** had 23,26-lactone unit. Anti-inflammatory assay revealed that compounds **3** and **5** exhibited significant inhibitory activities against NO production in LPS-induced RAW 264.7 cells and cyclooxygenase (COX-2).

## 1. Introduction

**Figure 1:**
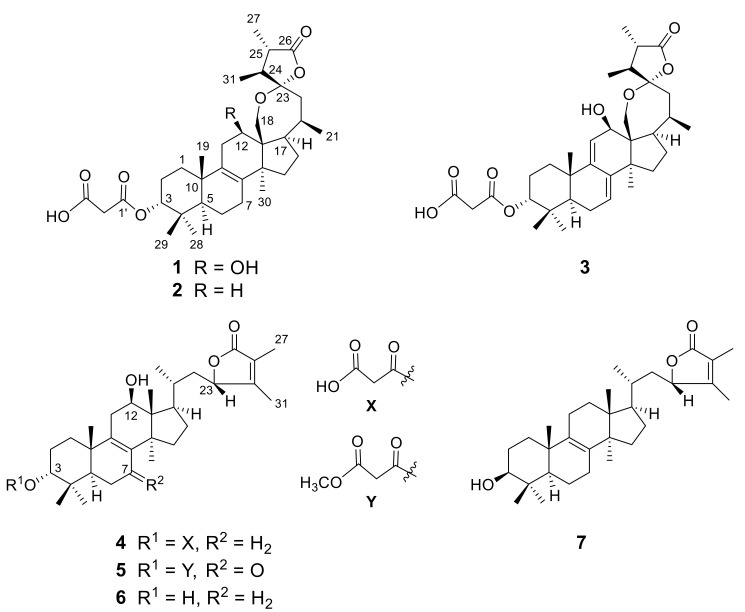
Structures of compounds **1−8**.

24-methyl-lanostane triterpenoids are a class of compounds present in most fungal species of the family Polyporaceae, which has attracted wide attention among the natural product chemistry community due to their diversity structure and bioactivities [1]. *Fomes officinalis* (Vill. Ex Fr.) Ames (Polyporaceae) is a perennial wood-decay fungus, distributed in the Northern regions of China and in the Pacific Northwest United States, Canada, and Europe [2,3]. *F. officinalis* was traditionally used to treat cough and asthma in the Chinese Uygur Medicine prescription [2]. Previous phytochemical investigation on this species led to isolate a variety of lanostane triterpenoids, which exhibited broad biological activity such as cytotoxicity, anti-inflammation, antibacterial, and anti-leukaemia [4,5,6,7]. In our search for bioactive compounds from Xinjiang medicinal materials, eight new 24-methyl-lanostane triterpenoids, officimalonic acids A−H, were previously isolated and identified from the fruiting bodies of *F. officinalis* by our group [8]. In the current study, further chemical investigation on this species led to isolate another seven undescribed lanostane triterpenoids, named officimalonic acids I−O (**1**–**7**) (Figure 1). The inhibitory activity of the isolates against NO production in LPS-induced RAW264.7 cells were also evaluated. Herein, the isolation, structural elucidation, and the evaluation of anti-inflammatory activity, were present.

## 2. Results and Discussion

Compound **1** was isolated as colorless amorphous solid; its molecular formula was identified as C_34_H_50_O_8_ by HR-ESI-MS ion peak at *m/z* 609.3382 [M + Na]^+^ (calculated for C_34_H_50_O_8_Na, 609.3403) (See Supplementary Materials). The IR spectrum of 1 showed the absorption bands for hydroxyl (3545 cm^−1^) and carboxyl (1779 and 1732 cm^−1^), which indicated the presence of a strained lactone unit. The ^1^H-NMR spectrum (Table 1) of **1** exhibited four tertiary methyl groups at *δ*_H_ 1.07, 1.03, 0.96 and 0.90 (each 3H, s), three secondary methyls at *δ*_H_ 1.18 (3H, d, *J* = 6.8 Hz), 1.15 (3H, d, *J* = 7.1 Hz) and 0.99 (3H, d, *J* = 6.3 Hz), two oxygenated methine protons at *δ*_H_ 4.68 (brs, H-3) and 4.21 (t, *J* = 7.1 Hz, H-12), as well as a oxygenated methylene protons at 3.91 (1H, d, *J* = 14.3 Hz) and 3.88 (1H, d, *J* = 14.3 Hz). The ^13^C-NMR (Table 2) spectra indicated the presence of 33 carbons sorted to seven methyls, nine methylenes, seven methines and 10 quaternary carbons (three of which are carbonyls). It is worth to mention that the NMR data of CH_2_-2′ were not observed when they were acquired in CD_3_OD, which is consistent with that of officimalonic acid A [8].

**Figure 2:**
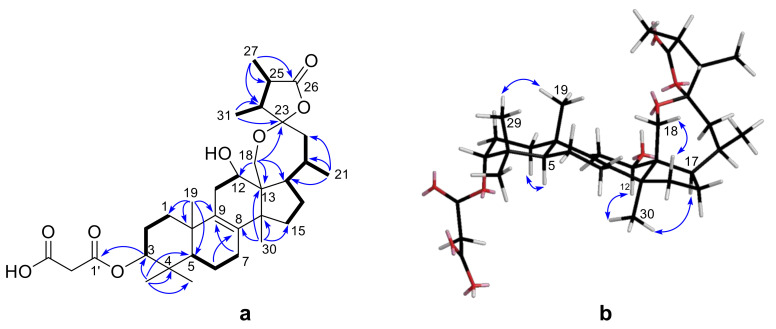
(**a**) Key ^1^H−^1^H COSY (▬) and selected HMBC correlations (H→C) of **1**; (**b**) selected NOESY correlations (H↔H) of **1**.

Further analysis of 2D NMR data (^1^H-^1^H COSY, HSQC, HMBC) revealed that the structure of **1** was similar with that of officimalonic acid C, previously isolated from this species by our group [8]. The only difference between them is the absence of C-7 carbonyl in **1**. The ^1^H-^1^H COSY correlations (Figure 2a) of H-5/H-6/H-7, and the HMBC correlations (Figure 2a) from H-6 to C-8 and from H-7 to C-9, confirmed the assignment. The relative configurations of **1** were determined by the NOESY correlations (Figure 2b) and coupling constant. The H-3 was established as β-configuration by its broad singlet-like signal. The NOESY correlation between H-12 and H_3_-30 indicated they were α-configuration. Considering the biogenetic relationship, the absolute configurations of **1** were tentatively assigned as those of officimalonic acid C. Thus, the structure of compound **1** was elucidated as depicted, given the trivial name officimalonic acid I.

Compound **2** was determined to have the molecular formula C_34_H_50_O_7_ by HR-ESI-MS at *m/z* 571.3598 [M + H]^+^ (calculated for C_34_H_51_O_7_, 571.3635), 16 mass less than that of **1**. The ^1^H and ^13^C-NMR (Table 1 and Table 2) data of **2** showed very similar results with those of **1**. The major difference was that instead of a methylene signal, the signals of oxygenated methine (CH-12) were missed in **2**, suggesting that compound **2** is one less hydroxyl than **1**. The ^1^H-^1^H COSY correlations of H-11/H-12 and the HMBC correlation from H_2_-18 to C-12, supported the assignment. The structure of **2** was thus elucidated as depicted, named officimalonic acid J.

Compound **3** had a molecular formula C_34_H_48_O_8_, which was determined by the HR-ESI-MS at *m/z* 607.3218 [M + Na]^+^ (calculated for C_34_H_48_O_8_Na, 607.3247). Analysis of ^1^H and ^13^C-NMR (Table 1 and Table 2) data of **3** indicated that it had a similar structure with **1**. The obvious differences between them were the presence of two double bonds (Δ^7^ and Δ^9(11)^) in **3** instead of one double bond (Δ^8^) present in **1**. The two olefinic protons signals at *δ*_H_ 5.57 (brs, H-7) and 5.40 (brs, H-11) supported the assignment. The ^1^H-^1^H COSY correlations of H-5/H-6/H-7 and H-11/H-12, together with the HMBC correlations from H-7 to C-9, and from H-11 to C-8, confirmed the above assignment. Therefore, the structure of **3** was elucidated as depicted, named officimalonic acid K.

**Figure 3:**
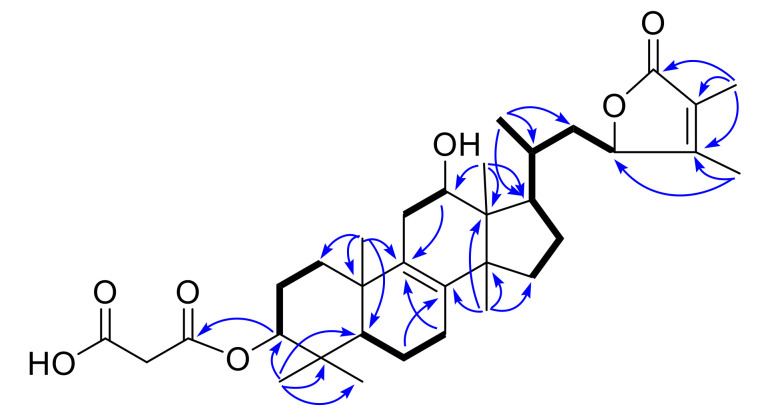
Key ^1^H−^1^H COSY (▬) and selected HMBC correlations (H→C) of **4**.

Compound **4** was obtained as a colorless amorphous solid; its HR-ESI-MS displayed a quasi-molecular ion peak at *m/z* 615.3512 [M + HCOO]^−^, corresponding to the molecular formula C_34_H_50_O_7_. Analysis of ^1^H and ^13^C-NMR (Table 1 and Table 2) data of **4** revealed it shared the close structure similarity with that of officimalonic acid B, previously isolated from the same species [8]. The difference between these two compounds is the absence of a carbonyl group signal and the presence of a methylene in **4**. The ^1^H-^1^H COSY correlations (Figure 3) of H-5/H-6/H-7, and the HMBC correlations (Figure 3) from H-6 to C-8 and from H-7 to C-9, indicated that compound **4** possess methylene at C-7 in **4** instead of a carbonyl group in officimalonic acid B. The structure of **3** was thus elucidated as depicted, gave a trivial name officimalonic acid L.

Compound **5** had a molecular formula C_35_H_50_O_8_, which was established by its HR-ESI-MS at *m/z* 621.3384 [M + Na]^+^ (calculated for C_35_H_50_O_8_Na, 621.3403). The ^1^H and ^13^C-NMR (Table 1 and Table 2) data of **5** showed almost the same as those of officimalonic acid B, the only difference being the presence of one more methoxyl signal in **5**. The methoxy group was attached to C-3′ of malonate half-ester by the HMBC correlation from *δ*_H_ 3.68 (3H, s) to C-3′ (*δ*_C_ 167.4). Therefore, the structure of **5** was determined as depicted, named officimalonic acid M.

Compound **6** had a molecular formula of C_31_H_48_O_4_ as determined on the basis of HR-ESI-MS at *m/z* 507.3427 [M + Na]^+^ (calculated for C_35_H_50_O_8_Na, 507.3450). Analysis of ^1^H and ^13^C-NMR (Table 1 and Table 2) data of **6** revealed it had a similar structure with that of **4**. The difference is that the signals for malonate half-ester at C-3 are not observed in **6**, correspondingly, its proton signal of H-3 is up-field shifted to *δ*_H_ 3.36 (brs), indicated that compound **6** had hydroxy group at C-3, rather than carboxyacetyloxy group at C-3. The HMBC correlations from H_3_-28 (or H_3_-29) to C-3, C-4, and C-5, supported the assignment. The structure of **6** was thus elucidated as depicted, given the trivial name officimalonic acid N.

Compound **7** had a molecular formula of C_31_H_48_O_3_ by HR-ESI-MS at *m/z* 469.3651 [M + H]^+^ (calculated for C_31_H_49_O_3_, 469.3682), 16 mass less than that of **6**. The ^1^H and ^13^C-NMR (Table 1 and Table 2) data of **7** showed very similar signals with those of **6**. The only two oxygenated methines signals [*δ*_H_ 3.22 (dd, *J* = 11.6, 4.4 Hz), *δ*_C_ 79.0; *δ*_H_ 4.71 (brd, *J* = 8.0 Hz), *δ*_C_ 83.2] were observed in **7**, suggesting that it had one less hydroxy group than that of **6**. Further analysis 2D NMR spectra permitted to determine the structure. The HMBC correlations from H_3_-28 (or H_3_-29) to C-3 (*δ*_C_ 79.0), C-4 and C-5, indicated there was a hydroxy group at C-3, while the HMBC correlations from H_3_-18 to C-12 (*δ*_C_ 30.9), C-13, C-14 and C-17, suggested hydroxyl at C-12 in **6** disappeared in **7**. Notably, coupling constant of H-3 [9] of compound **7** is different with that of compounds **1**–**6**, implying its stereochemistry is not β-orientation, which is confirmed by the NOESY correlation between H-3 and H-5. Therefore, the structure of **7** was determined as depicted, named officimalonic acid O.

All of the new compounds had their inhibitory activities tested against NO production in lipopolysaccharide (LPS)-induced RAW264.7 cells. Their cell viabilities were firstly examined by the CCK-8 method. As compounds **2** and **4** showed strong cytotoxic effects against RAW 264.7 cells at the concentration of 60 μM, they would not be conducted, following anti-inflammation evaluation. Except compounds **2** and **4**, other tested compounds showed NO inhibition lower than 60 μM. The results indicated that compounds **3** and **5** exhibited significant inhibitory activities against NO production in LPS-induced RAW 264.7 cells with IC_50_ values at 33.0 and 25.4 μM, compared to that of positive control dexamethasone (IC_50_ = 20.35 μM).

In addition, the cyclooxygenase (COX-2) inhibitory activities of these new compounds were evaluated using the in vitro assay kit [10]. Interestingly, the similar results were observed as those of NO production inhibition assay, only compounds **3** and **5** showed COX-2 inhibitory activities with IC_50_ values at 30.1 and 42.3 μM, compared to that of positive control naproxen (IC_50_ = 8.2 μM).

## 3. Materials and Methods

### 3.1. General Experimental Procedures

IR spectra were recorded on a Nicolet 380 FT-IR spectrometer. The optical rotations were measured on an Auto Pol IV automatic polarimeter (Rudolph Research, Flanders, NJ, USA) at room temperature. 1D and 2D NMR data were recorded on a Varian 400 MHz instrument with TMS as internal standard. HR-ESI-MS data were acquired using a Triple TOF 6600 mass spectrometer (AB Sciex, Framingham, MA, USA). Semi-preparative HPLC separations were performed on a Hitachi Chromaster system consisting of a 5110 pump, 5210 autosampler, 5310 column oven, 5430 diode array detector, and a Phenomenex Luna C18 column (250 × 10 mm, S-5 μm), all operated using EZChrom Elite software. All solvents were of ACS or HPLC grade, and were obtained from Tansoole (Shanghai, China), Sigma-Aldrich (St. Louis, MO, USA), respectively. Silica gel (300−400 mesh), C18 reverse-phased silica gel (150–200 mesh, Merck, city, country), and MCI gel (CHP20P, 75−150 μM, Mitsubishi Chemical Industries Ltd., Tokyo, Japan) were used for column chromatography (CC), and pre-coated silica gel GF254 plates (Qingdao Marine Chemical Plant, Qingdao, China) were used for TLC.

### 3.2. Fugnal Material

Fruiting bodies of *F. officinalis* were bought from Xinjiang Uyghur Medicine Hospital (Xinjiang, China), and identified by Prof. Xinping Yang (Institute of Applied Microbiology, Xinjiang Academy of Agricultural Sciences). A voucher specimen (FO-201411) is deposited in the Key Laboratory of Plant Resources and Chemistry of Arid Zone, Xinjiang Technical Institute of Physics and Chemistry, Chinese Academy of Sciences (Xinjiang, China).

### 3.3. Extraction and Isolation

Air-dried ground powder of *F. officinalis* fruiting bodies (1.0 kg) was extracted with MeOH (4.5 L × 3) by maceration at room temperature (seven days each time) to afford a crude methanol extract (575.9 g). The crude extract was suspended in distilled H_2_O and then extracted successively with EtOAc and *n*-BuOH. The EtOAc fraction (135.8 g) was then applied to a column of MCI gel (MeOH-H_2_O, 50:50 to 100:0, *v*/*v*) to yield five fractions (A−E). Fraction B (8.4 g) was then subjected to silica gel CC eluted with a CHCl_3_: MeOH (100:1 to 1:1 *v*/*v*) gradient to obtain seven fractions (B1−B7). Fraction B1 (1.3 g) was subjected to RP-18 silica gel CC (MeOH-H_2_O, 60/40 to 100:0, *v*/*v*) to give four sub-fractions (B1a−B1d). Purification of B1b (87.0 mg) by semi-preparative HPLC, eluting with MeOH-H_2_O (0–25 min: 70:30 to 78:22; 25–26 min: 78:22 to 100:0; 26–27 min: 100:0; 27–28 min: 100:0 to 70:30; 28–35 min: 70:30; *v*/*v*, 3 mL/min), yielded compounds **2** (6.4 mg) and **3** (7.5 mg). Fraction B3 (2.5 g) was separated on RP-18 silica gel CC (MeOH-H_2_O, 60/40 to 100:0, *v*/*v*) to give four sub-fractions (B3a−B3d). Sub-fraction B3a (562.4 mg) was subjected to silica gel CC eluted with a CHCl_3_: MeOH (100:1 to 1:1 *v*/*v*) gradient to obtain two fractions (B3a1−B3a2). Fraction B3a1 (172.7 mg) was purified by semi-preparative HPLC, eluting with isocratic MeOH-H_2_O (65:35, *v*/*v*, 3 mL/min) to yield the compound **1** (10.6 mg). Fraction D (16.8 g) was subjected to silica gel CC eluted with a CHCl_3_: MeOH (100:1 to 1:1 *v*/*v*) gradient to obtain three fractions (D1−D3). Fraction D1 (3.6 g) was separated on RP-18 silica gel CC (MeOH-H_2_O, 80/20 to 100:0, *v*/*v*) to give four sub-fractions (D1a−D1d). Sub-fraction D1b (2.31 g) was subjected to silica gel CC eluted with a CHCl_3_: MeOH (100:1 to 1:1 *v*/*v*) gradient to obtain seven fractions (D1b2−D1b7). Purification of D1b5 (149.2 mg) by silica gel CC eluted with a CHCl_3_: MeOH (60:1 to 50:1 *v*/*v*) gradient to yield compound **4** (12.3 mg). Fraction D3 (2.4 g) was subjected to a Sephadex LH-20 column eluted with MeOH to obtain three sub-fractions (D3a−D3c). Purification of D3a (1.8 g) by semi-preparative HPLC, eluting with MeOH-H_2_O (0–25 min: 83:17 to 85:15; 25–26 min: 85:15 to 100:0; 26–27 min: 100:0; 27–28 min: 100:0 to 83:17; 28–35 min: 83:17; *v*/*v*, 3 mL/min), yielded compounds **5** (6.3 mg) and **6** (34.2 mg). Fraction E (42.5 g) was subjected to silica gel CC eluted with a petroleum ether: EtOAc (50:1 to 1:1 *v*/*v*) gradient to obtain four fractions (E1−E4). Fraction E4 (3.4 g) was separated by silica gel CC eluted with a petroleum ether: EtOAc (20:1 to 0:1 *v*/*v*) gradient to obtain five fractions (E4a−E4e). Fraction E4c (1.4 g) was subjected to RP-18 silica gel CC (MeOH-H_2_O, 70:30 to 100:0, *v*/*v*) to give three sub-fractions (E4c1−E4c3). Purification of E4c3 (654.1 mg) by semi-preparative HPLC, eluting with MeOH-H_2_O (0-20 min: 90:10; 21–40 min: 90:10 to 94:6; 40–41 min: 94:6 to 100:0; 41–42 min: 100:0; 42–43 min: 100:0 to 90:10; 43–50 min: 90:10; *v*/*v*, 3 mL/min), yielded compounds **7** (6.4 mg).

*Officimalonic acid I(**1**)*: white amorphous powder; [α]^20^_D_ = −45 (*c* 0.100, MeOH); IR ν_max_ 3544, 2956, 1779, 1732, 1456, 1376, 1142, 1062 cm^−1^; ^1^H-NMR and ^13^C-NMR data, see Table 1 and Table 2; HR-ESI-MS: *m/z* 609.3382 [M + Na]^+^ (calculated for C_34_H_50_O_8_Na, 609.3403).

*Officimalonic acid J(**2**)*: white amorphous powder; [α]^20^_D_ = −32 (*c* 0.05, MeOH); IR ν_max_ 3523, 2953, 1775, 1723, 1456, 1375, 1269, 1155, 918 cm^−1^; ^1^H-NMR and ^13^C-NMR data, see Table 1 and Table 2; HR-ESI-MS: *m/z* 571.3598 [M + H]^+^ (calculated for C_34_H_51_O_7_, 571.3635).

*Officimalonic acid K(**3**)*: white amorphous powder; [α]^20^_D_ = −20 (*c* 0.05, MeOH); IR ν_max_ 3400, 2933, 1780, 1683, 1455, 1376, 1207, 1140, 926 cm^−1^; ^1^H-NMR and ^13^C-NMR data, see Table 1 and Table 2; HR-ESI-MS: *m/z* 607.3218 [M + Na]^+^ (calculated for C_34_H_48_O_8_Na, 607.3247).

*Officimalonic acid L(**4**)*: white amorphous powder; [α]^20^_D_ = −6 (*c* 0.100, MeOH); IR ν_max_ 3392, 2948, 1733, 1594, 1456, 1374, 1207, 1033 cm^−1^; ^1^H-NMR and ^13^C-NMR data, see Table 1 and Table 2; HR-ESI-MS: *m/z* 615.3512 [M + HCOO]^-^ (calculated for C_35_H_51_O_9_, 615.3533).

*Officimalonic acid M(**5**)*: white amorphous powder; [α]^20^_D_ = −36 (*c* 0.05, MeOH); IR ν_max_ 3420, 2953, 1733, 1683, 1457, 1376, 1204, 1140, 1031 cm^−1^; ^1^H-NMR and ^13^C-NMR data, see Table 1 and Table 2; HR-ESI-MS: *m/z* 621.3384 [M + Na]^+^ (calculated for C_35_H_50_O_8_Na, 621.3403).

*Officimalonic acid N(**6**)*: white amorphous powder; [α]^20^_D_ = −17 (*c* 0.05, MeOH); IR ν_max_ 3446, 2923, 1734, 1683, 1457, 1375, 1042 cm^−1^; ^1^H-NMR and ^13^C-NMR data, see Table 1 and Table 2; HR-ESI-MS: *m/z* 507.3427 [M + Na]^+^ (calculated for C_35_H_50_O_8_Na, 507.3450).

*Officimalonic acid O(**7**)*: white amorphous powder; [α]^20^_D_ = +6 (*c* 0.05, MeOH); IR ν_max_ 3260, 2930, 1745, 1683, 1447, 1370, 1087, 1030 cm^−1^; ^1^H-NMR and ^13^C-NMR data, see Table 1 and Table 2; HR-ESI-MS: *m/z* 469.3651 [M + H]^+^ (calculated for C_31_H_49_O_3_, 469.3682).

### 3.4. NO Production Inhibition Assay

Detection of accumulated nitrites was performed using Griess reagent (Beyotime, Shanghai, China) as described previously [8]. Briefly, the RAW264.7 cells at approximately 1.5 × 10^4^ cells/well were incubated for another 24 h, then cultured with or without LPS (1.0 μg/mL) in the presence or absence of the test compounds or positive control (10 μL) for another 24 h, the culture supernatant (50 μL) and Griess reagent (100 μL) were mixed to measure the optical density at a wavelength of 540 nm. Dexamethasone was used as positive control.

### 3.5. Cyclooxygenase (COX-2) Inhibition Assay

The COX-2 inhibitory activity study was carried out using ovine recombinant COX-2 enzyme by a COX colorimetric inhibitor screening assay kit from Cayman Chemical Co. (Ann Arbor, MI, USA). The assay was performed following the protocol provided by the manufacturer of assay kit. Non-selective inhibitor, naproxen, was used as a positive control. The COX inhibitory activity was expressed as % inhibition and was calculated as following Equation:

%inhibition = (ΔAbs _control_ − ΔAbs _sample_) × 100/ ΔAbs _control_

## 4. Conclusions

In the current study, seven new 24-methyl-lanostane triterpenoids were isolated and identified from the methanolic extract of the fruiting bodies of *Fomes* *officinalis*. For the structure characteristics, compounds **1**−**3** possessed an unusual spirostructure moiety at C-23, while compounds **4**−**7** had 23,26-lactone unit. The anti-inflammatory bioassay indicated that compounds **3** and **5** exhibited significant inhibitory activities against NO production in LPS-induced RAW 264.7 cells and COX-2, which may account for the traditional use for the inflammatory related disease.

## Figures and Tables

**Table 1:** ^1^H-NMR spectroscopic data (400 MHz) for compounds **1−7** (*δ* in ppm, *J* in Hz).

Position	1 ^a^	2 ^b^	3 ^a^	4 ^a^	5 ^a^	6 ^a^	7 ^b^
1	1.55 (2H, m)	1.46 (m)	1.75 (2H, m)	1.50 (2H, m)	1.66 (2H, m)	1.65 (m)	1.71 (m)
		1.34 (m)				1.45 (m)	1.19 (m)
2	1.98 (m)	1.87 (m)	2.00 (m)	1.93 (m)	2.07 (m)	1.98 (m)	1.63 (m)
	1.72 (m)	1.68 (m)	1.80 (m)	1.71 (m)	1.79 (m)	1.60 (m)	1.55 (m)
3	4.68 (brs)	4.72 (brs)	4.73 (brs)	4.71 (brs)	4.71 (brs)	3.36 (brs)	3.22 (dd, 11.6, 4.4)
4							
5	1.53 (m)	1.41 (m)	1.53 (m)	1.51 (m)	2.04 (m)	1.57 (m)	1.02 (m)
6	1.65 (m)	1.55 (m)	2.07 (2H, m)	1.63 (m)	2.51 (dd, 16.2, 14.6)	1.65 (m)	1.65 (m)
	1.53 (m)	1.41 (m)		1.51 (m)	2.26 (dd, 16.2, 3.2)	1.54 (m)	1.49 (m)
7	2.04 (2H, m)	1.97 (2H, m)	5.57 (brs)	2.04 (2H, m)			2.01 (2H, m)
11	2.75 (m)	1.62 (2H, m)	5.40 (brs)	2.52 (dd, 18.1, 7.8)	2.88 (dd, 20.6, 8.0)	2.57 (dd, 17.9, 8.0)	1.95 (m)
	2.04 (m)			1.80 (m)	2.11 (m)	1.83 (m)	1.28 (m)
12	4.21 (t, 7.7)	2.05 (m)	4.35 (brs)	4.07 (t, 7.8)	4.10 (t, 7.5)	4.08 (t, 8.1)	1.68 (m)
		1.68 (m)					1.20 (m)
15	1.71 (m)	1.49 (m)	1.70 (m)	1.73 (m)	2.04 (m)	1.78 (m)	1.66 (m)
	1.37 (m)	1.33 (m)	1.52 (m)	1.21 (m)	1.84 (m)	1.23 (m)	1.18 (m)
16	1.87 (m)	2.04 (2H, m)	1.94 (m)	1.87 (m)	1.87 (m)	1.90 (m)	1.99 (2H, m)
	1.76 (m)		1.78 (m)	1.51 (m)	1.54 (m)	1.54 (m)	
17	2.39 (dd, 8.8, 8.1)	1.88 (m)	2.44 (m)	2.03 (m)	1.99 (m)	2.05 (m)	1.60 (m)
18	3.91 (d, 14.3)	3.53 (d, 12.8)	3.76 (d, 13.6)	0.76 (3H, s)	0.74 (3H, s)	0.78 (3H, s)	0.70 (3H, s)
	3.88 (d, 14.3)	3.34 (d, 12.8)	3.66 (d, 13.6)				
19	1.07 (3H, s)	0.94 (3H, s)	1.09 (3H, s)	1.06 (3H, s)	1.27 (3H, s)	1.05 (3H, s)	0.97 (3H, s)
20	2.62 (m)	2.08 (m)	2.60 (m)	2.12 (m)	2.24 (m)	2.16 (m)	1.66 (m)
21	0.99 (3H, d, 6.3)	0.89 (3H, d, 6.6)	1.00 (3H, d, 6.8)	1.14 (3H, d, 6.9)	1.15 (3H, d, 6.8)	1.15 (3H, d, 7.3)	1.04 (3H, d, 6.4)
22	2.63 (m)	2.56 (m)	2.61 (m)	2.17 (m)	2.19 (m)	2.19 (brd, 14.8)	1.87 (m)
	1.73 (m)	1.49 (m)	1.72 (m)	1.13 (m)	1.14 (m)		1.20 (m)
23				4.88 (overlapped)	4.88 (overlapped)	4.88 (brd, 8.1)	4.71 (brd, 8.0)
24	2.11 (m)	1.92 (m)	2.10 (m)				
25	2.47 (m)	2.46 (m)	2.40 (m)				
27	1.15 (3H, d, 7.1)	1.14 (3H, d, 7.0)	1.12 (3H, d, 6.7)	1.75 (3H, s)	1.76 (3H, s)	1.77 (3H, s)	1.78 (3H, s)
28	0.90 (3H, s)	0.85 (3H, s)	0.90 (3H, s)	0.90 (3H, s)	0.89 (3H, s)	0.94 (3H, s)	0.98 (3H, s)
29	0.96 (3H, s)	0.90 (3H, s)	1.02 (3H, s)	0.95 (3H, s)	1.04 (3H, s)	0.87 (3H, s)	0.79 (3H, s)
30	1.03 (3H, s)	0.94 (3H, s)	0.99 (3H, s)	0.96 (3H, s)	0.97 (3H, s)	0.95 (3H, s)	0.88 (3H, s)
31	1.18 (3H, d, 6.8)	1.19 (3H, d, 6.7)	1.15 (3H, d, 6.8)	2.00 (3H, s)	2.01 (3H, s)	2.01 (3H, s)	1.93 (3H, s)
2′	n.b.	3.42 (2H, s)	n.b.	n.b.	n.b.		
3′-OMe					3.68 (3H, s)		

*^a^* in CD_3_OD; *^b^* in CDCl_3_; n.b., not observed.

**Table 2:** ^13^C NMR spectroscopic data (100 MHz) for compounds **1–7.**

Position	1*^a^*	2*^b^*	3*^a^*	4*^a^*	5*^a^*	6*^a^*	7*^b^*
1	30.6	30.7	30.3	30.7	29.5	30.1	35.3
2	22.6	23.1	22.4	22.7	22.2	25.3	27.9
3	79.2	80.4	79.1	79.2	78.2	75.2	79.0
4	36.4	36.8	36.2	36.4	36.4	37.1	38.8
5	45.2	45.3	44.0	45.2	45.4	43.9	50.3
6	17.5	17.8	22.4	17.6	35.7	17.8	18.2
7	25.2	25.8	122.2	25.4	199.6	25.5	26.5
8	133.3	133.1	139.5	133.8	137.7	133.5	134.2
9	136.0	135.9	146.9	135.7	167.2	136.1	134.5
10	36.6	36.9	36.9	36.5	39.5	36.5	37.0
11	33.6	20.1	121.7	33.2	35.0	33.2	28.4
12	74.9	23.0	77.0	72.2	70.9	72.4	30.9
13	50.0	47.2	49.9	48.9	49.3	48.9	44.6
14	50.4	48.7	48.2	51.8	49.8	51.8	49.8
15	31.5	30.9	31.8	30.8	32.0	30.8	30.8
16	20.2	20.4	20.1	23.9	23.7	23.8	20.9
17	48.4	48.6	49.0	50.4	49.3	50.4	50.6
18	65.6	63.7	65.0	9.8	10.0	9.8	15.6
19	18.2	19.2	21.6	18.0	17.0	18.1	19.1
20	30.0	27.8	29.8	32.3	31.7	32.3	35.4
21	21.7	22.9	21.8	22.7	22.1	21.8	19.9
22	38.5	38.8	38.4	37.5	37.2	37.5	39.3
23	110.1	110.6	109.9	84.5	84.6	84.6	83.2
24	49.9	50.8	49.9	162.2	162.1	162.2	159.9
25	41.7	42.2	41.6	122.0	122.0	122.0	123.1
26	179.1	179.4	179.0	175.7	175.6	175.7	174.7
27	11.6	12.8	11.6	6.9	6.83	6.82	8.5
28	26.8	27.6	26.9	26.9	26.0	27.3	27.8
29	20.8	21.7	21.4	20.9	20.3	21.3	15.4
30	24.8	25.3	25.6	23.2	23.6	23.1	24.2
31	11.4	12.7	11.3	10.9	10.9	10.9	12.3
1′	166.8	166.9	170.2	170.0	166.0		
2′	n.b.	40.8	n.b.	n.b.	n.b.		
3′	168.8	170.6	173.7	173.1	167.4		
3′-OMe				−	51.3		

*^a^* in CD_3_OD; *^b^* in CDCl_3_; n.b., not observed.

## Supplementary Materials

The following are available online. Figure S1: ^1^H-NMR spectrum of officimalonic acid I (**1**) in CD_3_OD. Figure S2: ^13^C-NMR spectrum of officimalonic acid I (**1**) in CD_3_OD. Figure S3: HSQC spectrum of officimalonic acid I (**1**) in CD_3_OD. Figure S4: ^1^H-^1^H COSY spectrum of officimalonic acid I (**1**) in CD_3_OD. Figure S5: HMBC spectrum of officimalonic acid I (**1**) in CD_3_OD. Figure S6: NOESY spectrum of officimalonic acid I (**1**) in CD_3_OD. Figure S7: IR spectrum of officimalonic acid I (**1**) in CD_3_OD. Figure S8: HR-ESI-MS spectrum of officimalonic acid I (**1**). Figure S9: ^1^H-NMR spectrum of officimalonic acid J (**2**) in CDCl_3_. Figure S10: ^13^C-NMR spectrum of officimalonic acid J (**2**) in CDCl_3_. Figure S11: HR-ESI-MS spectrum of officimalonic acid J (**2**). Figure S12: ^1^H-NMR spectrum of officimalonic acid K (**3**) in CD_3_OD. Figure S13: ^13^C-NMR spectrum of officimalonic acid K (**3**) in in CD_3_OD. Figure S14: HR-ESI-MS spectrum of officimalonic acid K (**3**). Figure S15: ^1^H-NMR spectrum of officimalonic acid L (**4**) in CD_3_OD. Figure S16: ^13^C-NMR spectrum of officimalonic acid L (**4**) in in CD_3_OD. Figure S17: HR-ESI-MS spectrum of officimalonic acid L (**4**). Figure S18: ^1^H-NMR spectrum of officimalonic acid M (**5**) in CD_3_OD. Figure S19: ^13^C-NMR spectrum of officimalonic acid M (**5**) in CD_3_OD. Figure S20: HR-ESI-MS spectrum of officimalonic acid M (**5**). Figure S21: ^1^H-NMR spectrum of officimalonic acid N (**6**) in CD_3_OD. Figure S22: ^13^C-NMR spectrum of officimalonic acid N (**6**) in CD_3_OD. Figure S23: HR-ESI-MS spectrum of officimalonic acid N (**6**). Figure S24: ^1^H-NMR spectrum of officimalonic acid O (**7**) in CDCl_3_. Figure S25: ^13^C-NMR spectrum of officimalonic acid O (**7**) in CDCl_3_. Figure S26: HR-ESI-MS spectrum of officimalonic acid O (**7**).
