# Supplementary material for: Lanostane Triterpenoids from the Fruiting Bodies of Fomes officinalis and Their Anti-Inflammatory Activities
Source: Molecules. 2020 Oct 19;25(20):4807. doi: 10.3390/molecules25204807 (PMC7587970; doi:10.3390/molecules25204807)

Lanostane triterpenoids from the fruiting bodies of *Fomes officinalis* and their anti-inflammatory activities

Jianxin Han,[1,2,†] Wei Liu,[2,3,†] Miaomiao Li,[3] Yanpei Gu,[1] Ying Zhang,[1,*] and Tao Yuan [2,3,*]

[1]*Department of Food Science and Nutrition, School of Biosystems Engineering and Food Science, Zhejiang Key Laboratory for Agro-Food Processing, Zhejiang University, Hangzhou 310058, China; jianxin@zju.edu.cn (J.H.), happygyp@126.com (Y.G.)*

[2]*The Key Laboratory of Plant Resources and Chemistry of Arid Zone, and State Key Laboratory of Xinjiang Indigenous Medicinal Plants Resource Utilization, Xinjiang Technical Institute of Physics and Chemistry, Chinese Academy of Sciences, Urumqi 830011, China; ucasliuwei@126.com (W.L.)*

[3]*The Laboratory of Effective Substances of Jiangxi Genuine Medicinal Materials, College of Life Sciences, Jiangxi Normal University, Nanchang 330022, China; jxnulmm@163.com (M.L.)*

*Corresponding author: yzhang@zju.edu.cn (Y.Z.), tyuan@jxnu.edu.cn (T.Y.)

[†]These authors contributed equally to this work.

Supporting Information

S1. ^{1}H NMR spectrum of officimalonic acid I (**1**) in CD_3OD

S2. ^{13}C NMR spectrum of officimalonic acid I (**1**) in CD_3OD

S3. HSQC spectrum of officimalonic acid I (**1**) in CD_3OD

S4. ^{1}H-^{1}H COSY spectrum of officimalonic acid I (**1**) in CD_3OD

S5. HMBC spectrum of officimalonic acid I (**1**) in CD_3OD

S6. NOESY spectrum of officimalonic acid I (**1**) in CD_3OD

S7. IR spectrum of officimalonic acid I (**1**)

S8. HRESIMS spectrum of officimalonic acid I (**1**)

S9. ^{1}H NMR spectrum of officimalonic acid J (**2**) in $CDCl_3$

S10. ^{13}C NMR spectrum of officimalonic acid J (**2**) in $CDCl_3$

S11. HRESIMS spectrum of officimalonic acid J (**2**)

S12. ^{1}H NMR spectrum of officimalonic acid K (**3**) in CD_3OD

S13. ^{13}C NMR spectrum of officimalonic acid K (**3**) in in CD_3OD

S14. HRESIMS spectrum of officimalonic acid K (**3**)

S15. ^{1}H NMR spectrum of officimalonic acid L (**4**) in CD_3OD

S16. ^{13}C NMR spectrum of officimalonic acid L (**4**) in in CD_3OD

S17. HRESIMS spectrum of officimalonic acid L (**4**)

S18. ^{1}H NMR spectrum of officimalonic acid M (**5**) in CD_3OD

S19. ^{13}C NMR spectrum of officimalonic acid M (**5**) in CD_3OD

S20. HRESIMS spectrum of officimalonic acid M (**5**)

S21. ^{1}H NMR spectrum of officimalonic acid N (**6**) in CD_3OD

S22. ^{13}C NMR spectrum of officimalonic acid N (**6**) in CD_3OD

S23. HRESIMS spectrum of officimalonic acid N (**6**)

S24. ^{1}H NMR spectrum of officimalonic acid O (**7**) in $CDCl_3$

S25. ^{13}C NMR spectrum of officimalonic acid O (**7**) in $CDCl_3$

S26. HRESIMS spectrum of officimalonic acid O (**7**)

S1. ^{1}H NMR spectrum of officimalonic acid I (**1**) in CD_3OD

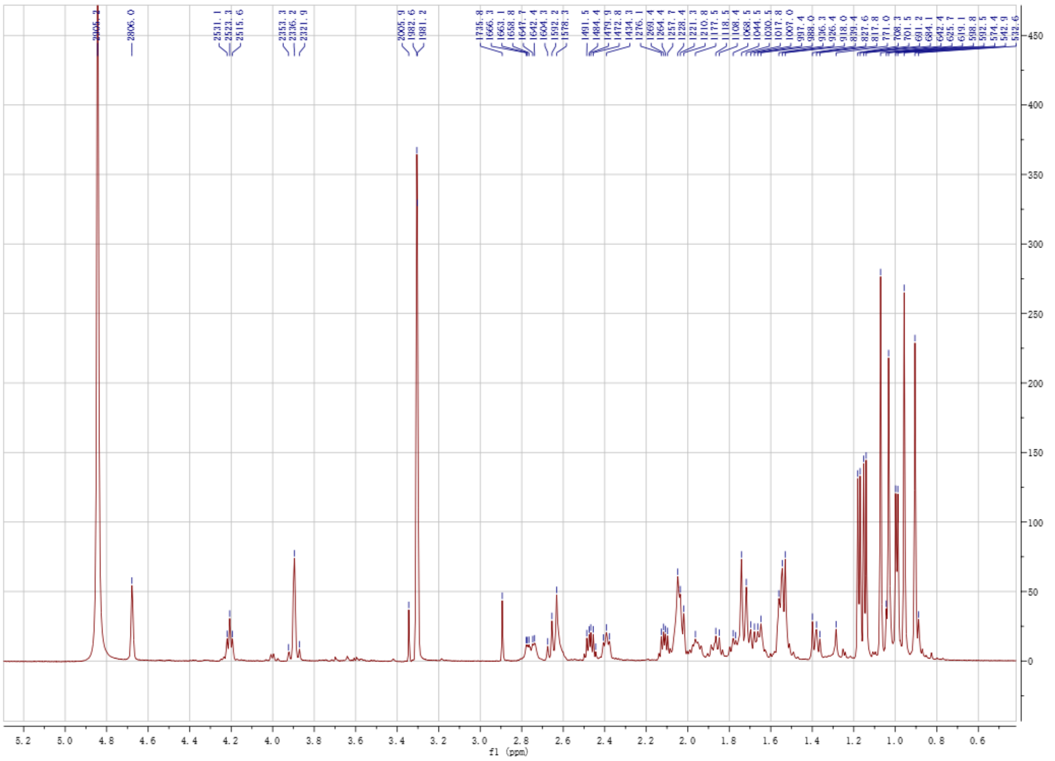

S2. ^{13}C NMR spectrum of officimalonic acid I (**1**) in CD_3OD

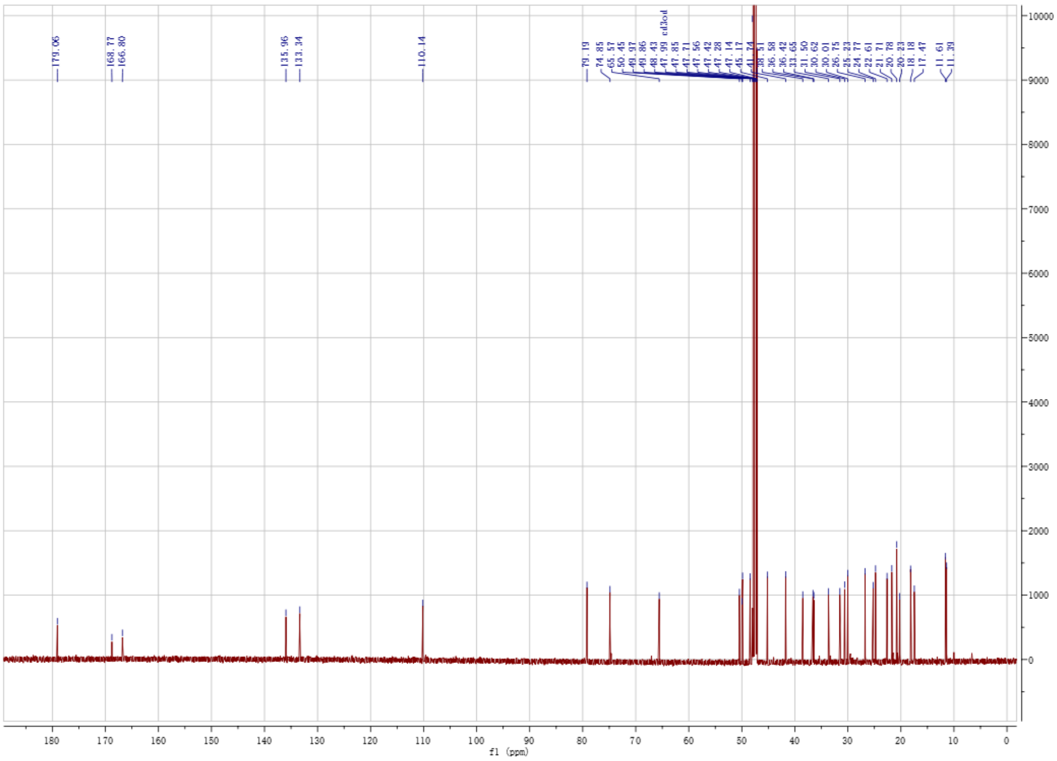

S3. HSQC spectrum of officimalonic acid I (**1**) in CD_3OD

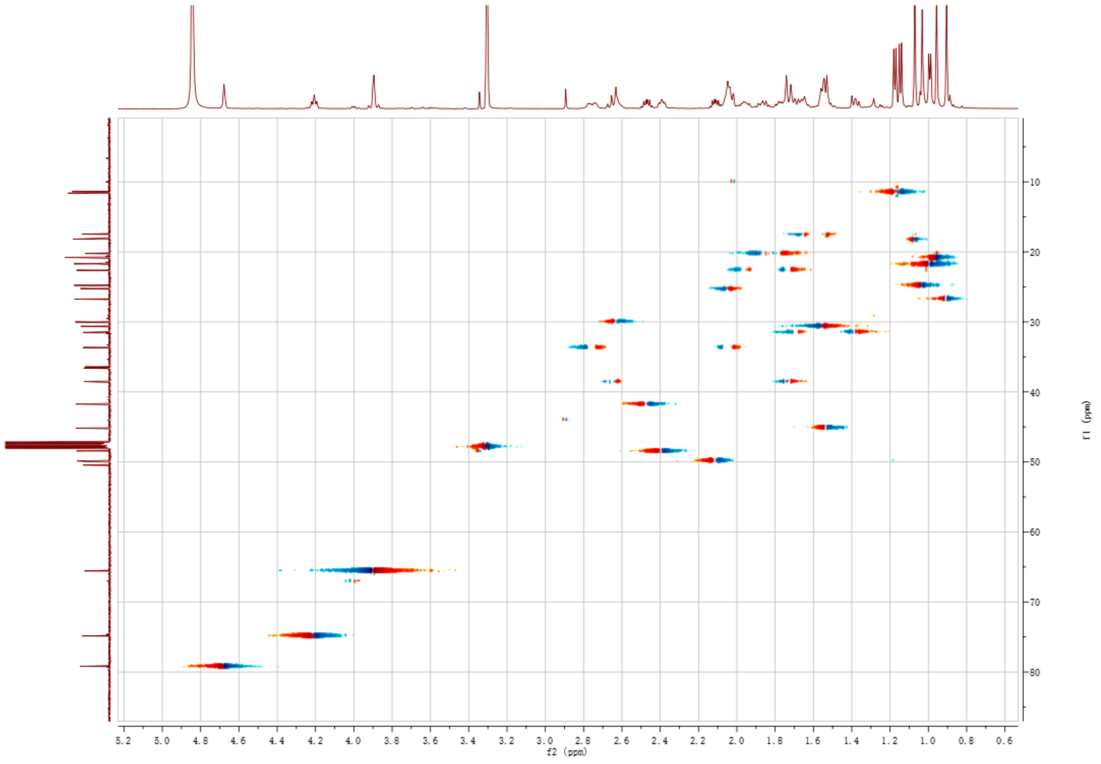

S4. ^1H-^1H COSY spectrum of officimalonic acid I (**1**) in CD_3OD

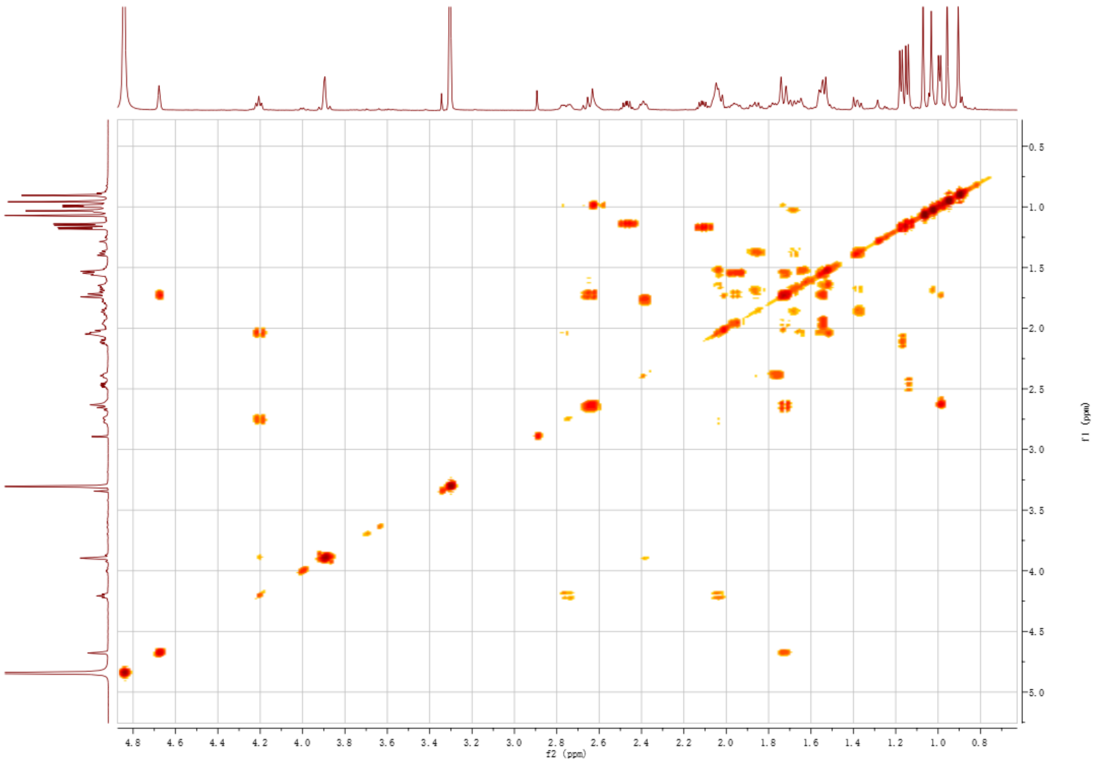

S5. HMBC spectrum of officimalonic acid I (**1**) in CD_3OD

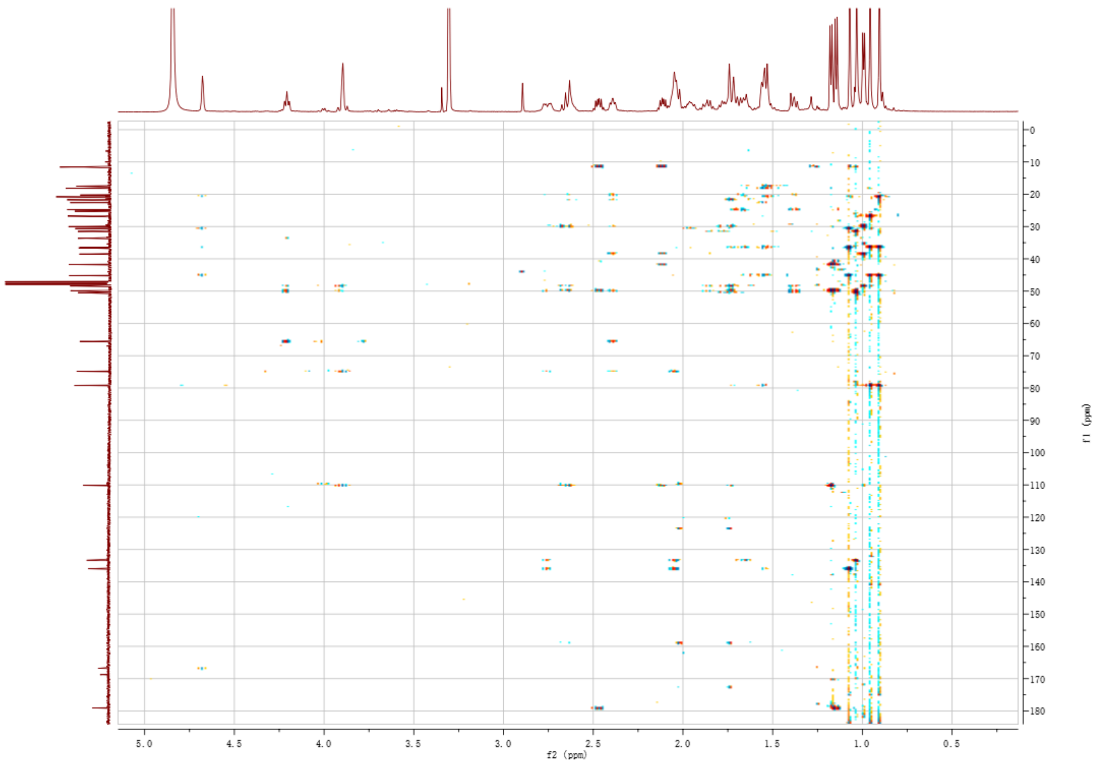

S6. NOESY spectrum of officimalonic acid I (**1**) in CD_3OD

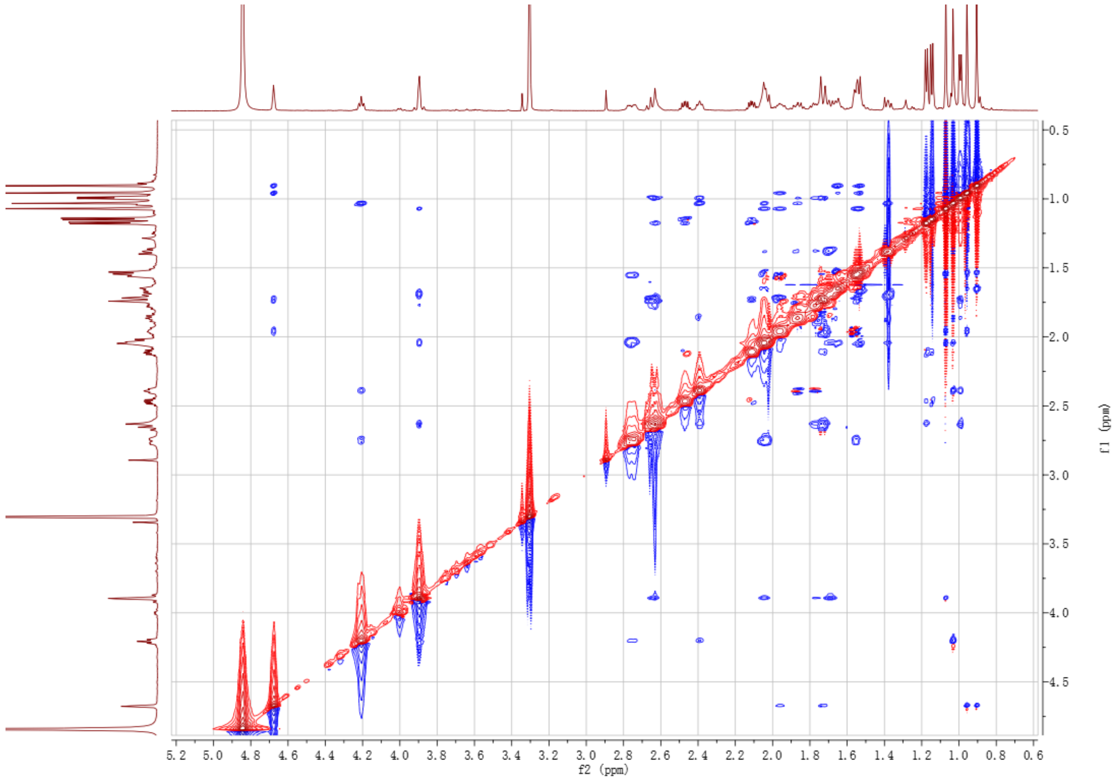

S7. IR spectrum of officimalonic acid I (**1**) in CD_3OD

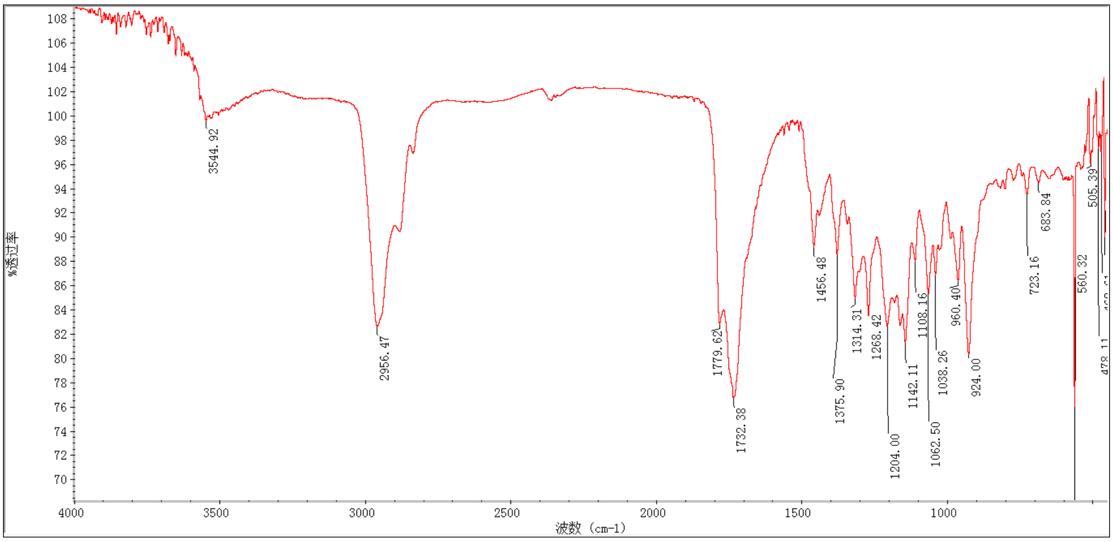

S8. HRESIMS spectrum of officimalonic acid I (**1**)

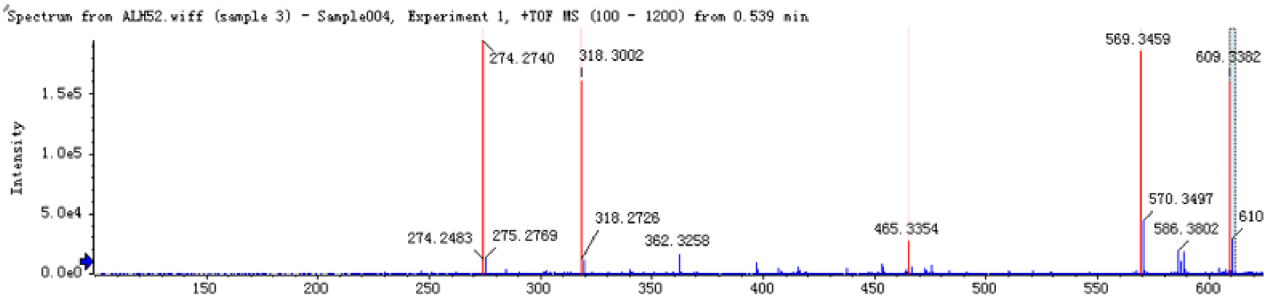

S9. ^{1}H NMR spectrum of officimalonic acid J (**2**) in $CDCl_3$

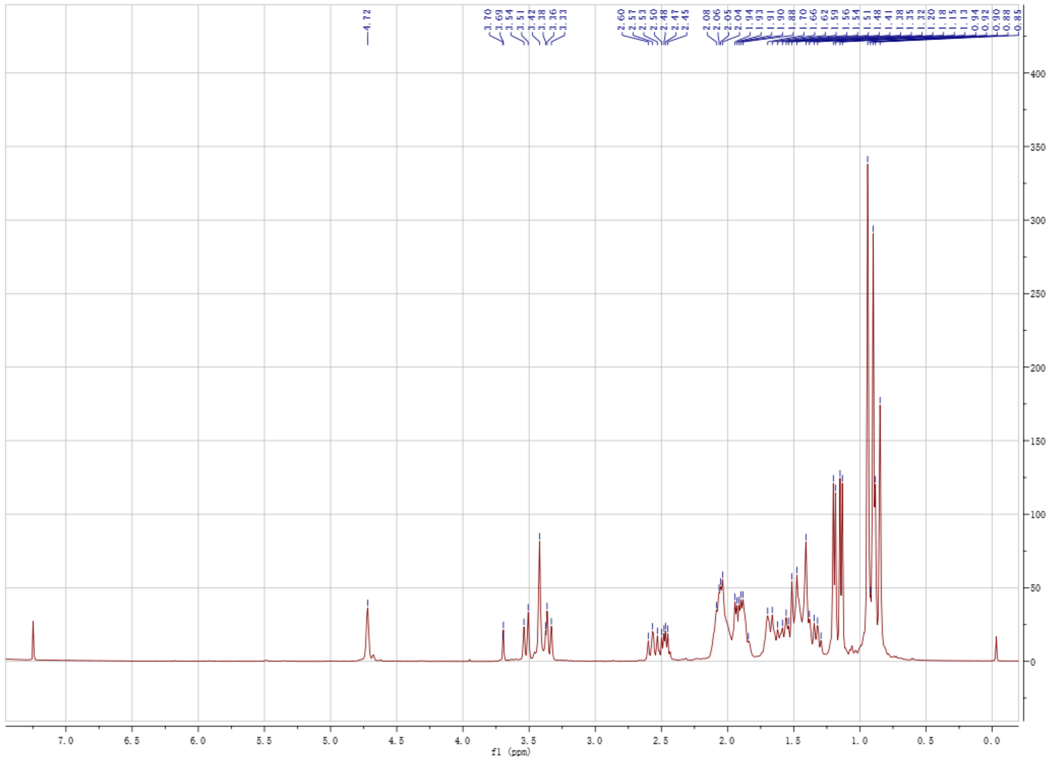

S10. ^{13}C NMR spectrum of officimalonic acid J (**2**) in $CDCl_3$

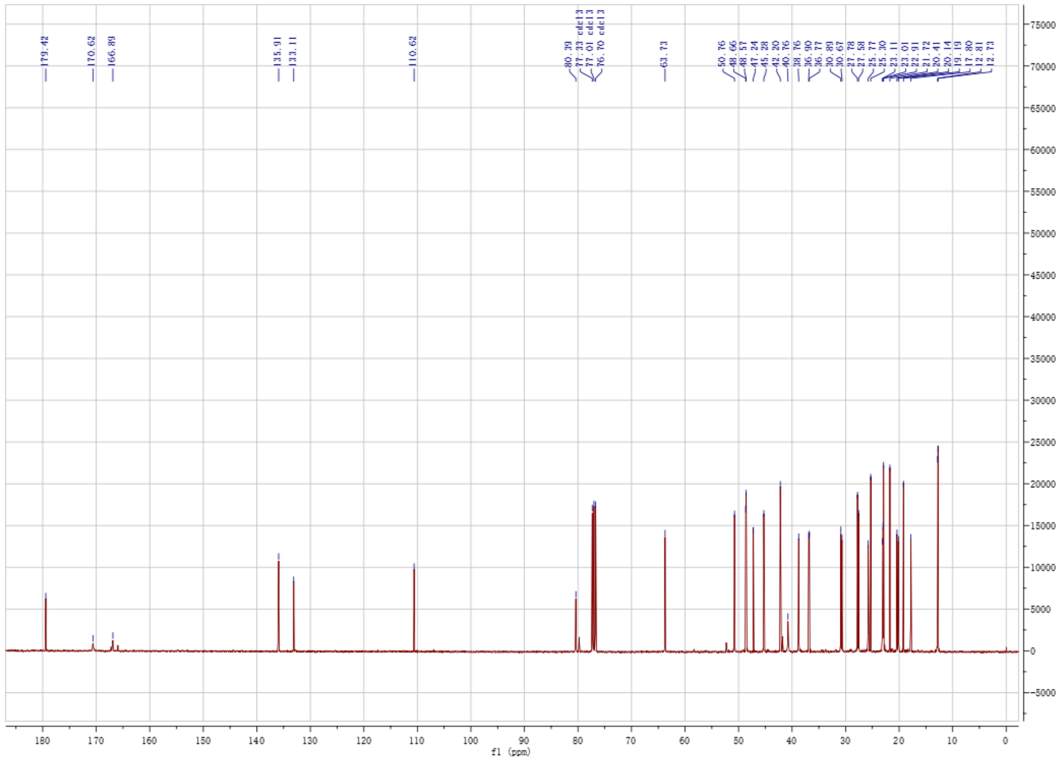

S11. HRESIMS spectrum of officimalonic acid J (**2**)

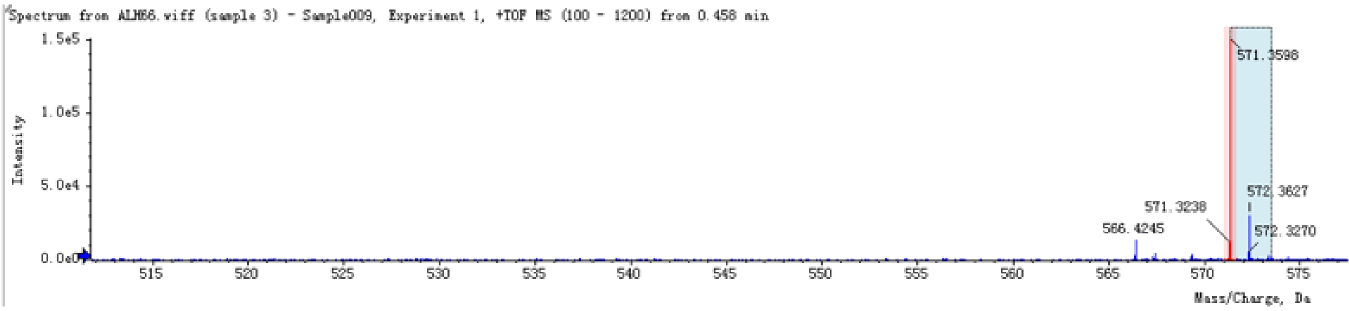

S12. ^{1}H NMR spectrum of officimalonic acid K (**3**) in CD_3OD

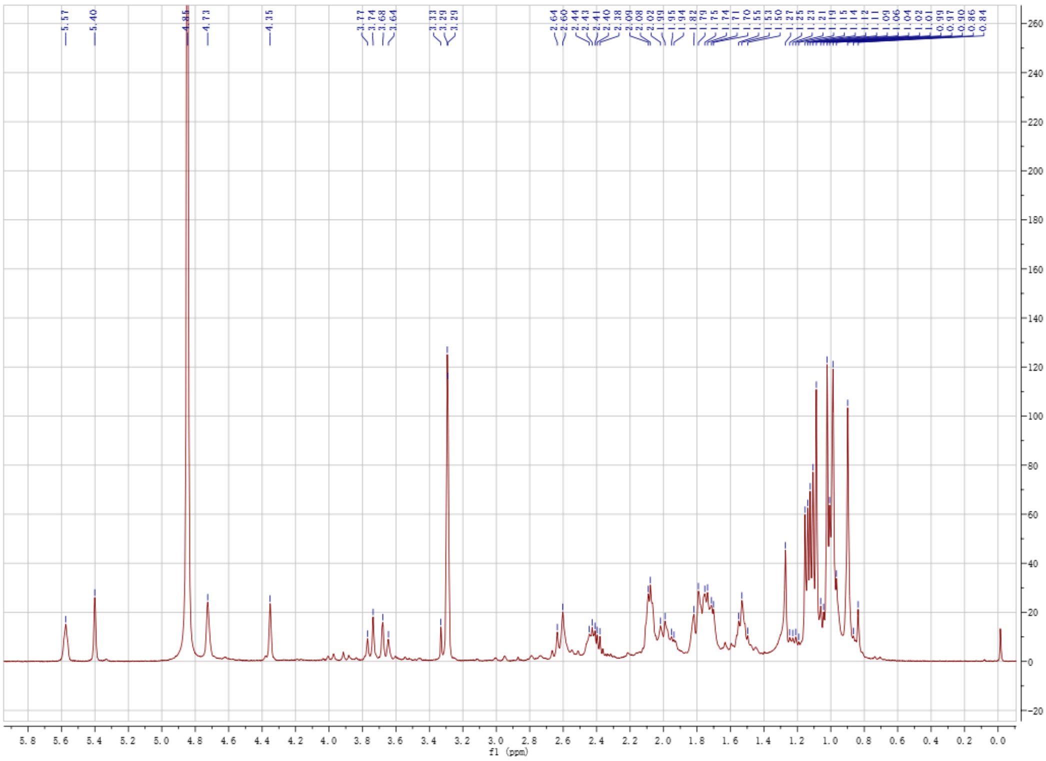

S13. ^{13}C NMR spectrum of officimalonic acid K (**3**) in in CD_3OD

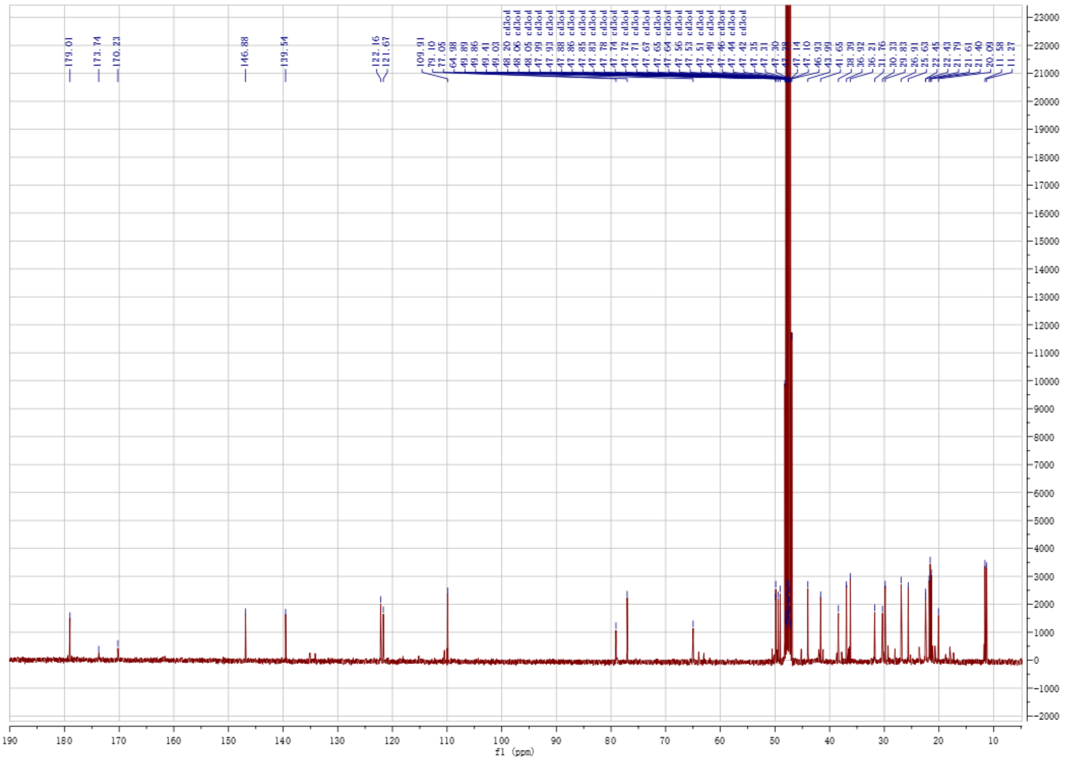

S14. HRESIMS spectrum of officimalonic acid K (**3**)

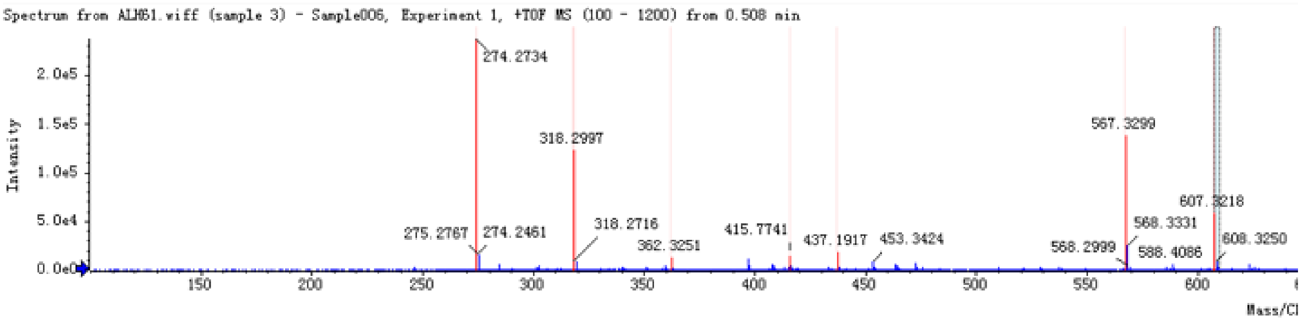

S15. ^{1}H NMR spectrum of officimalonic acid L (**4**) in CD_3OD

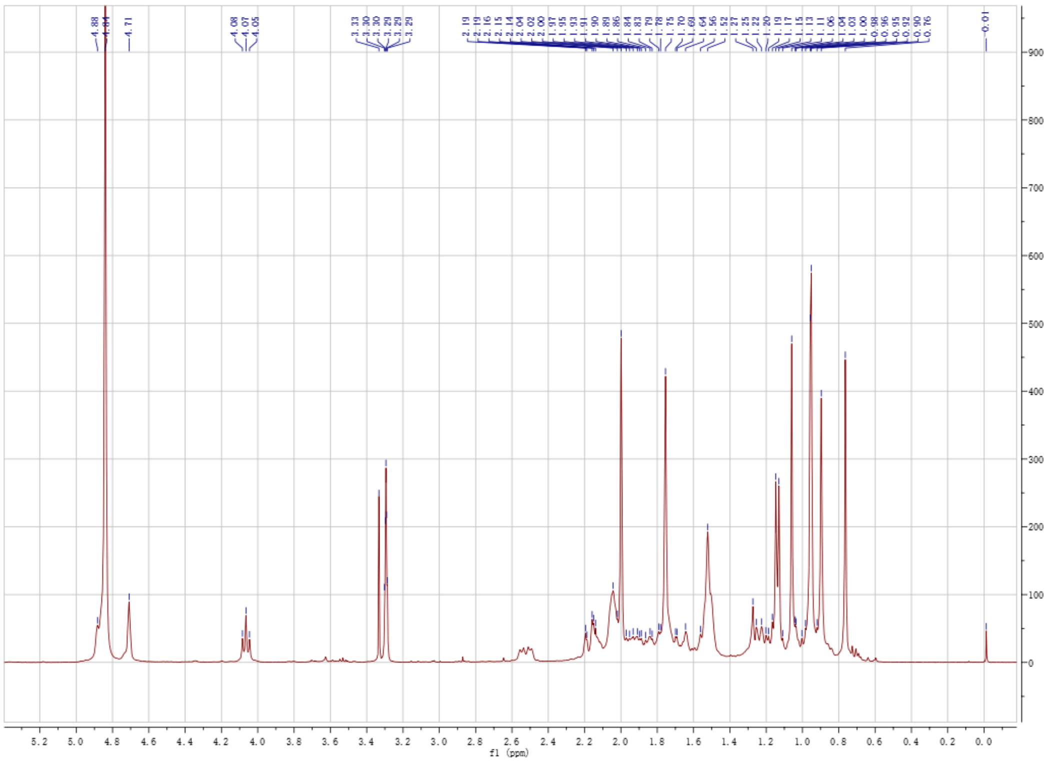

S16. ^{13}C NMR spectrum of officimalonic acid L (**4**) in in CD_3OD

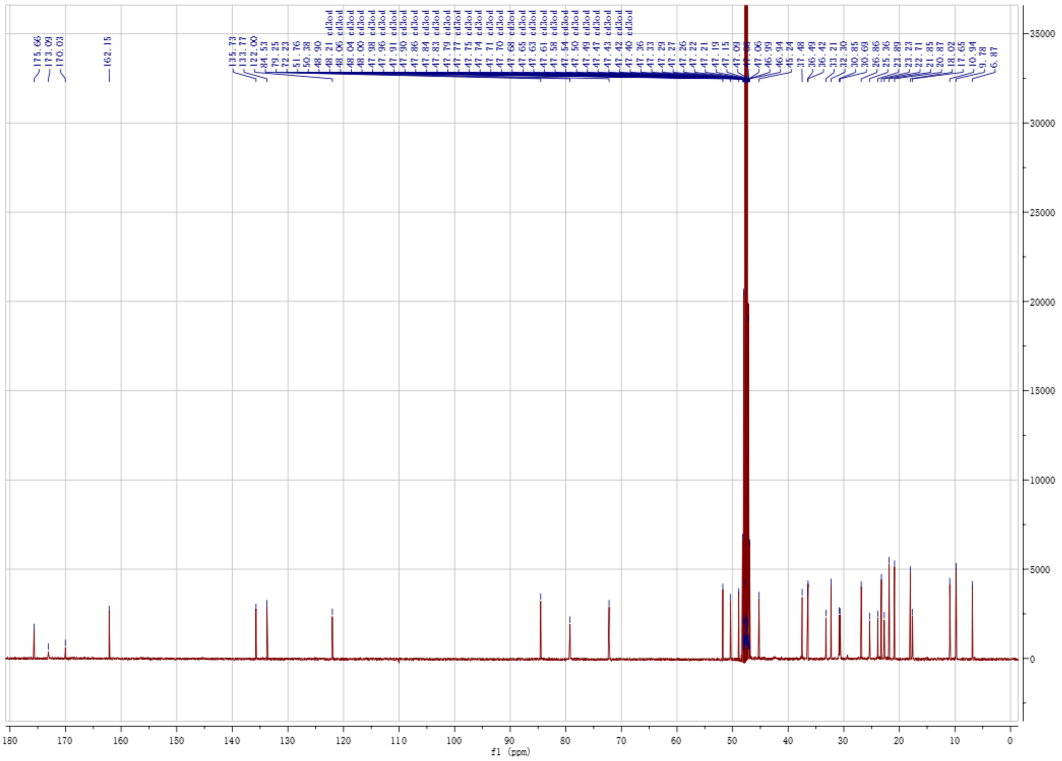

S17. HRESIMS spectrum of officimalonic acid L (**4**)

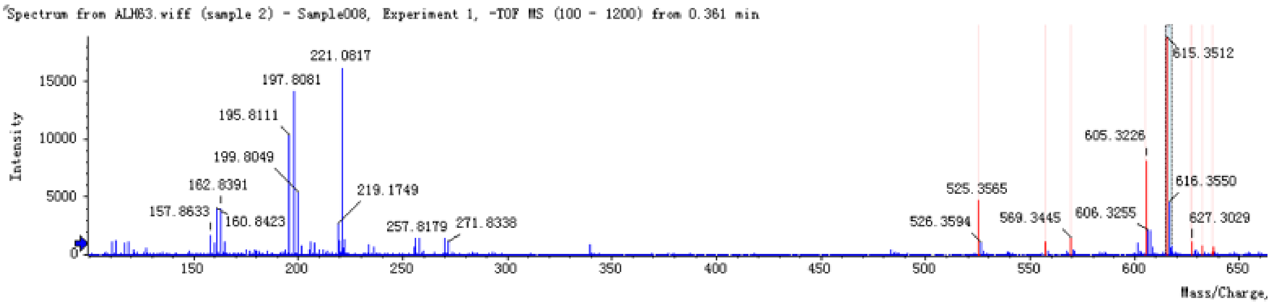

S18. ^{1}H NMR spectrum of officimalonic acid M (**5**) in CD_3OD

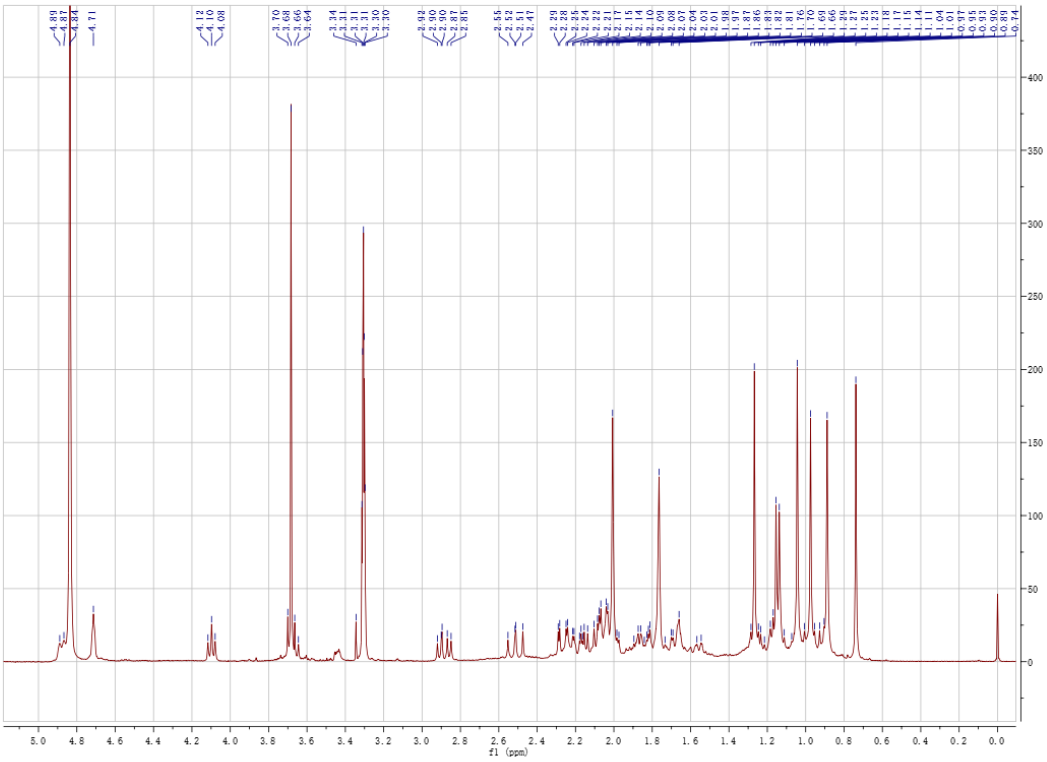

S19. ^{13}C NMR spectrum of officimalonic acid M (**5**) in CD_3OD

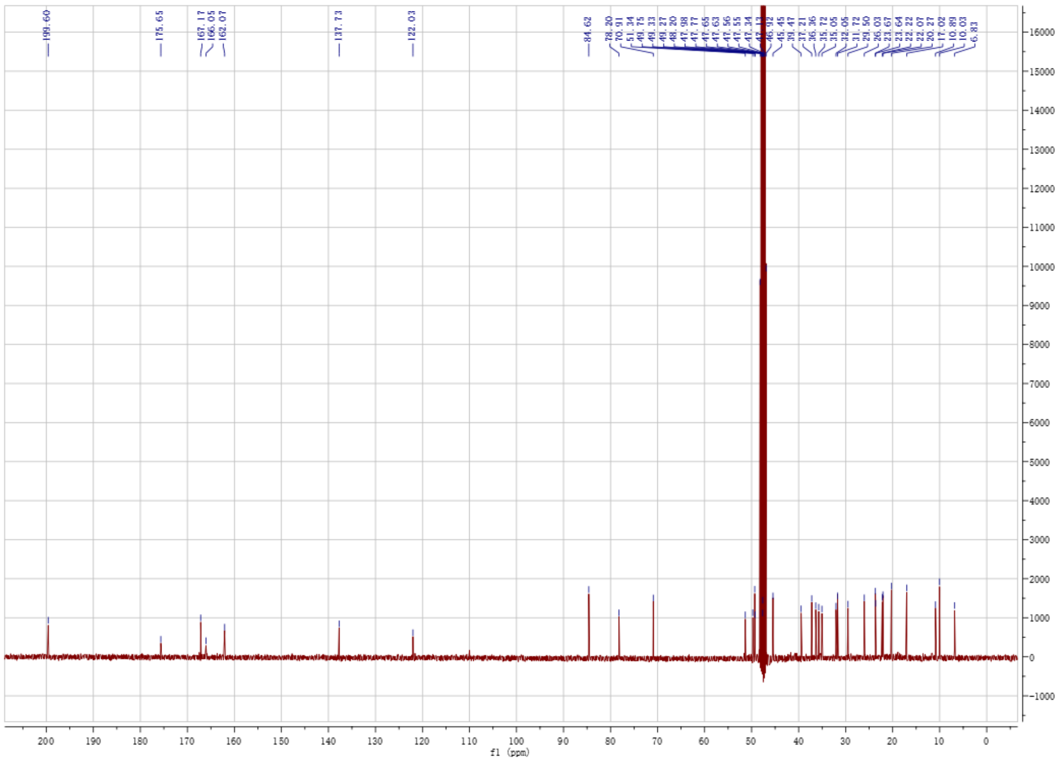

S20. HRESIMS spectrum of officimalonic acid M (**5**)

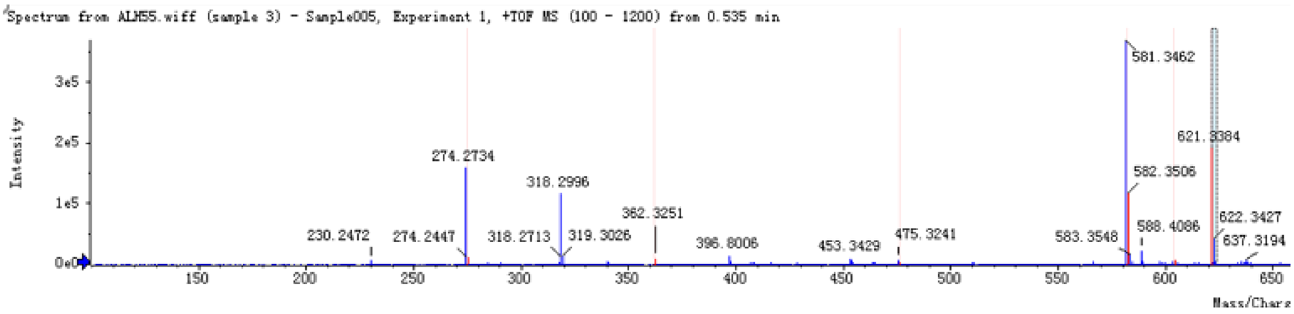

S21. ^{1}H NMR spectrum of officimalonic acid N (**6**) in CD_3OD

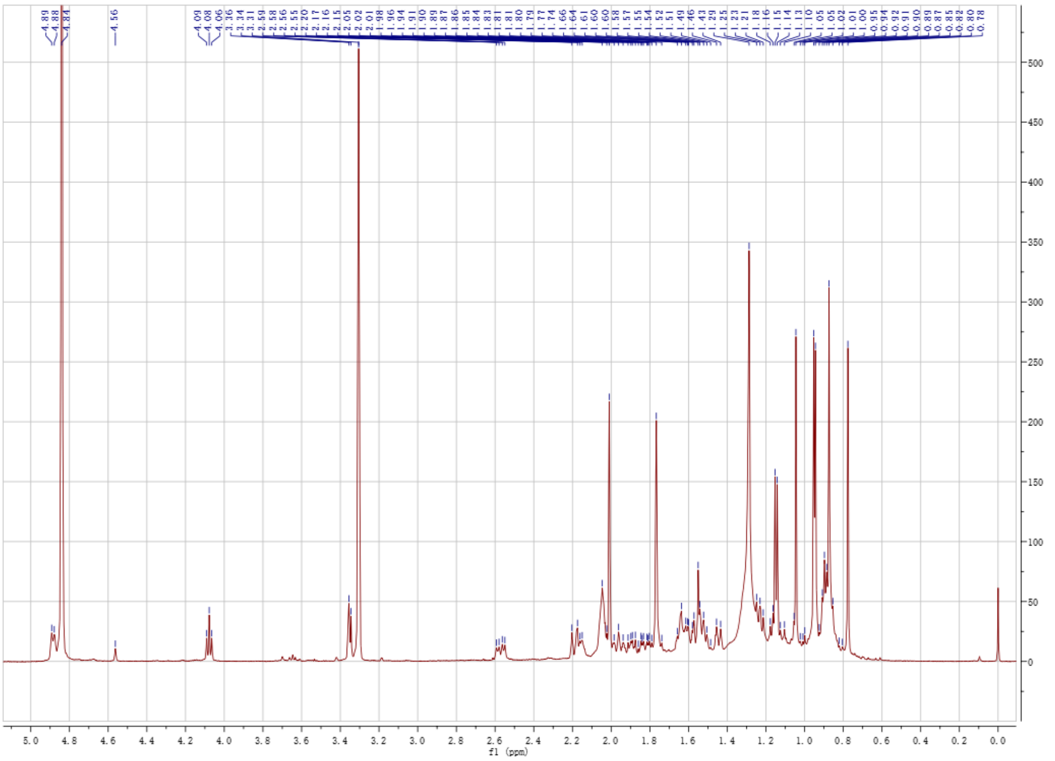

S22. ^{13}C NMR spectrum of officimalonic acid N (**6**) in CD_3OD

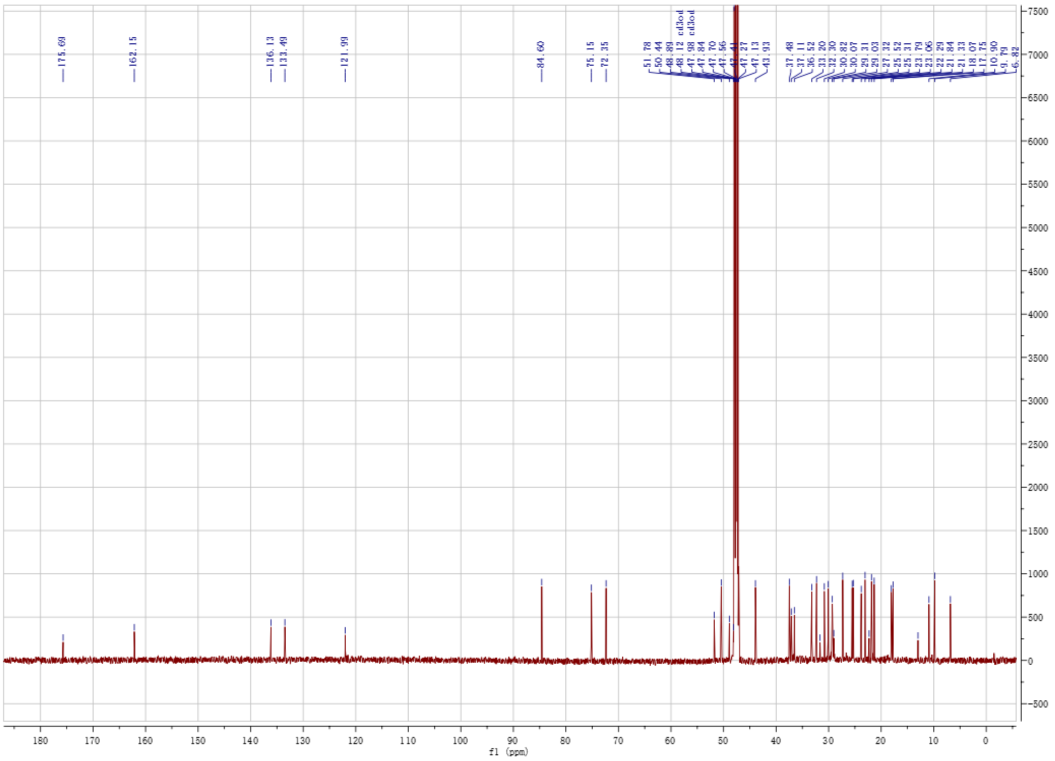

S23. HRESIMS spectrum of officimalonic acid N (**6**)

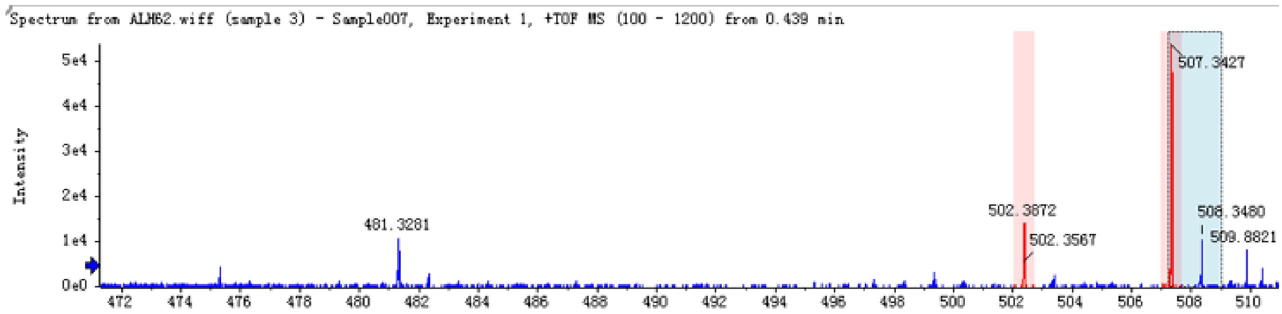

S24. ^{1}H NMR spectrum of officimalonic acid O (**7**) in $CDCl_3$

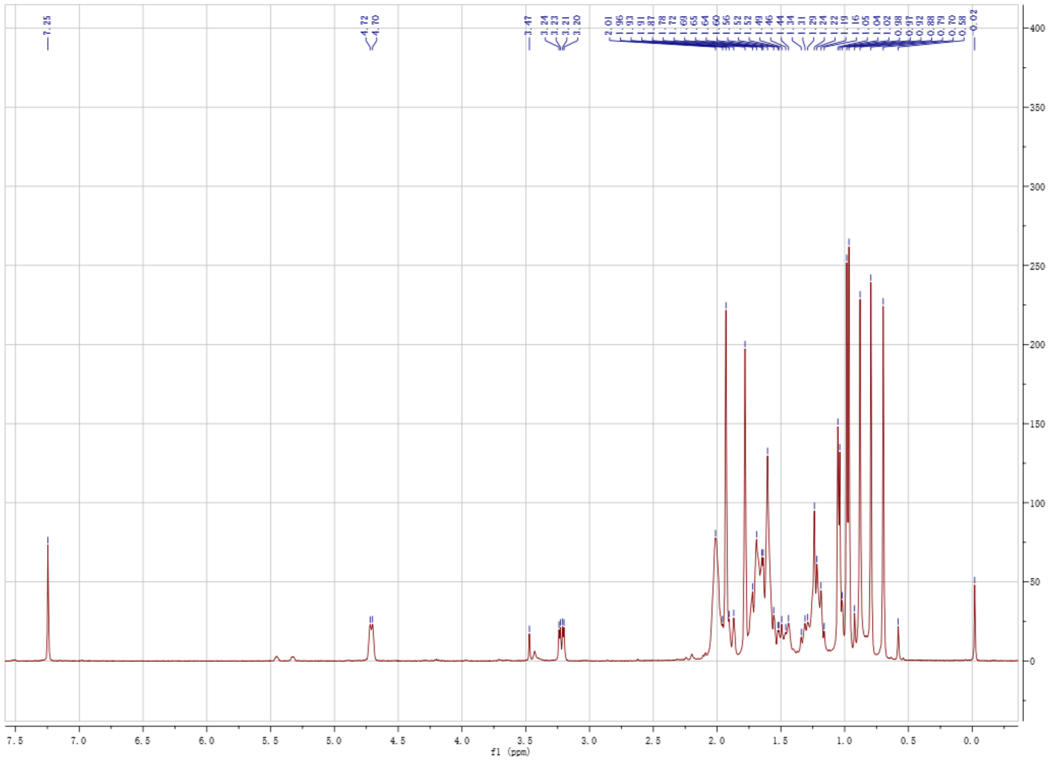

S25. ^{13}C NMR spectrum of officimalonic acid O (**7**) in $CDCl_3$

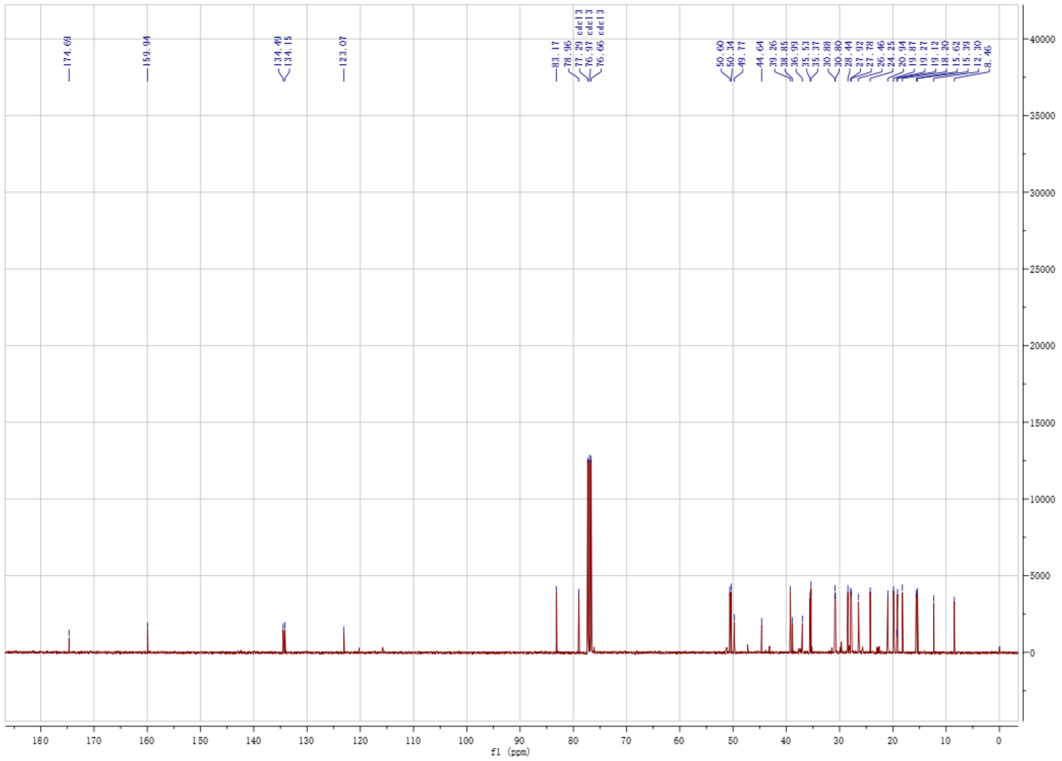

S26. HRESIMS spectrum of officimalonic acid O (**7**)

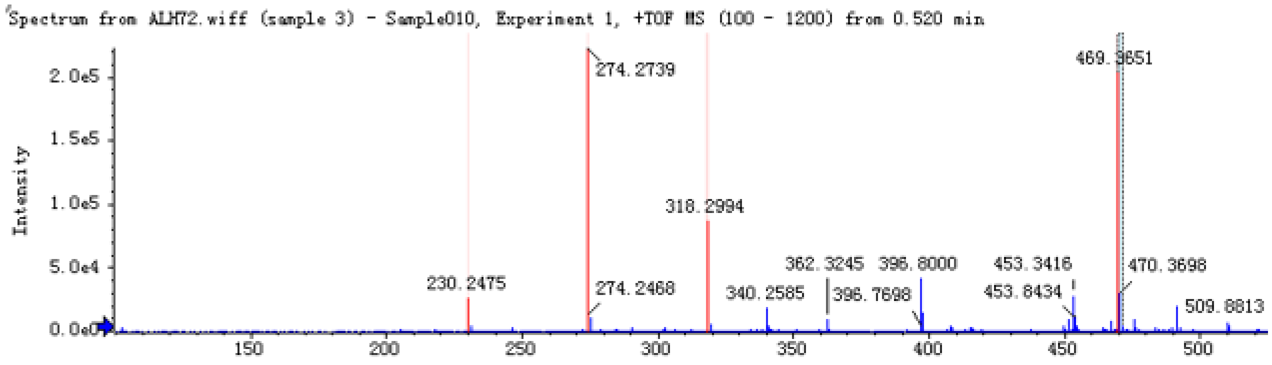

Supplement: Supplementary file 1 [file molecules-25-04807-s001.pdf]
